# Electrochemical Impedance Spectroscopy as a Tool for Electrochemical Rate Constant Estimation

**DOI:** 10.3791/56611

**Published:** 2018-10-10

**Authors:** Pavel Chulkin, Przemyslaw Data

**Affiliations:** ^1^Factulty of Chemistry, Department of Physical Chemistry and Technology of Polymers, Silesian University of Technology; ^2^Department of Physics, Durham University; ^3^Centre of Polymer and Carbon Materials of the Polish Academy of Sciences

**Keywords:** Chemistry, Issue 140, Electrochemical Impedance Spectroscopy, Redox Process, The Electrochemical Rate Constant, Emitters, Organic Electronics, Electrochemistry

## Abstract

Electrochemical impedance spectroscopy (EIS) was used for advanced characterization of organic electroactive compounds along with cyclic voltammetry (CV). In the case of fast reversible electrochemical processes, current is predominantly affected by the rate of diffusion, which is the slowest and limiting stage. EIS is a powerful technique that allows separate analysis of stages of charge transfer that have different AC frequency response. The capability of the method was used to extract the value of charge transfer resistance, which characterizes the rate of charge exchange on the electrode-solution interface. The application of this technique is broad, from biochemistry up to organic electronics. In this work, we are presenting the method of analysis of organic compounds for optoelectronic applications.

**Figure:**
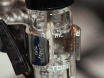


## Introduction

Redox rate of the electroactive compound is an important parameter characterizing its ability to undergo oxidation or reduction processes and predict its behavior in the presence of strong oxidizing or reducing agents or under applied potential. However, most of the electrochemical techniques are only able to qualitatively describe the kinetics of the redox process. Among various electrochemical techniques employed for redox active compounds, characterization cyclic voltammetry (CV) is the most prevailing method for quick and sufficient electrochemical characterization of various soluble species^1^,^2^,^3^. The CV technique has broad applications, *e.g.*, energy levels estimations^4^,^5^,^6^, the charge carriers analysis supported by spectroscopies^7^,^8^,^9^,^10^, up to surface modifications^11^,^12^,^13^. Like every method, CV is not perfect and to increase the applicability and quality of results, the connection with another spectroscopic technique is important. We already present several investigations where the electrochemical impedance spectroscopy (EIS) technique was employed^14^,^15^,^16^ but in this work, we intended to show step-by-step how to reinforce the CV technique by EIS.

The EIS output signal consists of two parameters: real and imaginary parts of impedance as functions of frequency^17^,^18^,^19^,^20^. It allows estimation of several parameters responsible for charge transfer through the electrode-solution interface: double layer capacitance, solution resistance, charge transfer resistance, diffusion impedance and other parameters depending on system investigated. Charge transfer resistance was an object of high attention since this parameter is directly related to the redox rate constant. Even though oxidation and reduction rate constants are estimated in solution, they may generally characterize the ability of a compound for charge exchange. EIS is considered to be an advanced electrochemical technique requiring profound mathematical understanding. Its main principles are described in modern electrochemistry literature^17^,^18^,^19^,^20^,^21^,^22^,^23^.

## Protocol

### 1. Basic Preparation of an Electrochemical Experiment

1. Prepare 4 mL of a working solution containing 0.1 mol∙L^−1^ Bu_4_NBF_4_ and 0.001 mol∙L^−1^ investigated organic compound by adding calculated amounts of solid powders into 4 mL of dichloromethane in a small vessel or a test tube. With 2,8-bis(3,7-dibutyl-10H-phenoxazin-10- yl)dibenzo[b,d]thiophene-S,S-dioxide (molar mass 802 g∙mol^−1^), weigh 3.208 mg of this compound and 0.1645 g of Bu_4_NBF_4_.
2. Fill a 3 mL electrochemical cell with 2 mL of solution using a pipette. The remaining portion of the solution will be needed later for impedance measurement and reproducing the results.
3. Polish a 1 mm diameter platinum working disc electrode (WE) for 30 s using a polishing cloth moistened by several drops of alumina slurry. Rub the flat surface of the disc electrode with a piece of cloth mounted on an immobile support (*e.g.* Petri dish) by applying moderate pressure.
4. Rinse the electrode with distilled water three times to remove alumina particles.
5. Anneal a counter electrode (CE, platinum wire) in a butane burner flame. Carefully put the platinum wire in a flame for less than 1 s and quickly remove when it starts reddening to avoid melting. ​NOTE: The CE surface area is not stipulated but must be much higher than the surface area of the working electrode. In this case, impedance of working electrode interface would have the major impact on the total system impedance and would permit excluding counter electrode impedance from consideration.
  1. Anneal a reference electrode (RE, silver wire) in butane burner flame in the same manner.
6. Put all three electrodes (working, counter and reference) into a cell avoiding mutual contact and connect to the corresponding potentiostat cables marked as WE, CE and RE. Insert a gas delivering tube connected with argon gas bottle for further deaeration.
7. Open the gas valve and deaerate solution by bubbling argon through the solution for 20 min. Close the gas valve before measurement.

### 2. Tentative Characterization by Cyclic Voltammetry (CVA)

1. Register the CVA of the working solution within a potential range from −2.0 V to +2.0 V and scan rate 100 mV∙s^−1^.
  1. Launch the program **Cyclic voltammetry** in the potentiostat software.
  2. Choose 0.0 V as initial potential value, −2.0 V as minimal potential, +2.0 V as maximal scanning potential, 100 mV∙s^−1^ as scanning rate. Other parameters are optional.
  3. Click the button **Start**. NOTE: A typical voltammogram is presented in Figure 1.
2. Determine the potential value from the CVA obtained. Note the potential values when maxima of positive (anodic peak) and negative (cathodic peak) current appear and calculate the average value.
3. Add 10 mg of ferrocene by spatula into the working solution and deaerate it by argon bubbling for 5 min. This is necessary for mixing and complete dissolution of the ferrocene added. NOTE: The ferrocene amount is not precise. However, adding less than 1 mg or more than 20 mg would complicate estimation of equilibrium potential.
4. Register the CVA of the working solution within the potential range from −1.0 V to +1.0 V and scan rate 100 mV∙s^−1^. A small reversible peak of ferrocene will appear as shown in Figure 1.
5. Determine the potential value of ferrocene reversible oxidation from the CVA obtained. Note the potential values when maxima of positive (anodic peak) and negative (cathodic peak) current appear and calculate the average value.
6. Put another portion of the solution prepared at step 1.1 into the cell and clean the electrodes by repeating the procedure described in 1.2-1.7.

### 3. Registration of Impedance Spectrum

NOTE: An example of the setup in software is shown in Figure 2; any other software or device also can be used. However, the setup arrangement may differ in different software, although the main principles remain the same. Use the EIS in a staircase mode, *i.e.* potentiostatic spectra are registered automatically one after another.

1. In the software, choose a potential range of 0.2 V covering the reversible peak in CVA. Example: A reversible oxidation peak was detected on CV at 0.7 V. The potential range for CV should be then from 0.6 V to 0.8 V. The spectra will be registered with the increment of 0.01 V, *i.e.* at 0.61 V, 0.62 V, etc.
2. Register the EIS automatic measurement procedure under following conditions advised.
  1. Enter the following input values: initial potential 0.6 V; finish potential 0.8 V; potential increment: 0.01 V; frequency range: from 10 kHz through 100 Hz; the number of frequencies in logarithmic scale: 20; wait for a time between the spectra: 5 s, ac voltage amplitude 10 mV, minimal 2 measures per frequency.
  2. Click the button **Start**. NOTE: In that case, 21 spectra, each containing 41 frequency points will be obtained. The typical set of automatically registered spectra is presented in Figure 3.

### 4. Analysis of Impedance Spectrum

1. Launch the program **EIS spectrum analyser**.
2. Download the spectrum by choosing **File | Open**.
3. In the right upper sub-window construct an EEC by using left/right mouse click choosing series or parallel connection and necessary element from the context menu: C - capacitor, R - resistor, W - Warburg element. Start from the simplest circuit (Figure 5**c**).
4. Choose initial minimal and maximal values for parameters by left-mouse-clicking table cells and entering values: C1 - from 1∙10^-7^ to 1∙10^-8^, R1 - from 2000 to 100, R2 - from 1000 to 100, Aw - from 50000 to 10000.
5. Fit the model by choosing **Model | Fit**. Repeat the procedure several times (usually about 5 times) until the calculated values no longer change. Parameter values are shown in a table in the upper left sub-window.
6. Check parameter errors shown in the last column of the table. If an error of a parameter exceeds 100%, that means that the parameter is not necessary for a circuit. In that case try another equivalent circuit. NOTE: If one tries to fit an experimental spectrum corresponding to the simple circuit (Figure 5**c**) by a more complicated circuit (Figure 5**a**), then errors of unnecessary additional parameters W and R3 would be considerably high.
7. Check the values of *r*^2^(parametric) and *r*^2^(amplitude) presented in the lower right sub-window. If they exceed limit 1∙10^−2^, repeat the procedures 4.2−4.5 using another equivalent electrical circuit (EEC) (Figure 5).
8. Repeat the procedure 4.1-4.7 for all the spectra registered
9. For each spectrum analyzed, write down the calculated value of charge transfer resistance and the corresponding potential that the spectrum was registered at.

### 5. Calculation of Redox Rate Constants

1. Put the values of the estimated inverse charge transfer resistance versus potential. A typical potential plot of inverse charge transfer resistance for the reversible process is presented in Figure 6.
  1. Open an empty sheet of spreadsheet software.
  2. Manually enter the values of potentials and corresponding values of reverse charge transfer resistance in columns A and B.
  3. Select the range A1:B21 and choose **Insert | Graph | Pointed** by mouse clicking in the task menu.
2. Plot the values of a theoretical function calculated by the formula (1) on the same plot. Use constant values: *F* = 96485 C∙mol^−1^, *c*_0_ = 0.01 mol∙^−1^, *z* = 1, *R* = 8.314 J∙mol^−1^∙K^−1^, α *= 0.5, T *- ambient temperature. Use the previously estimated value (3.1) of *E*_0_. 
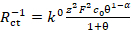
 (1) 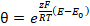 (2) where *R*_ct_^−1^ is inverse value of charge transfer resistance normalized by surface area; *z*- number of electrons transferred in one step (accepted being equal 1); *F*- Faraday constant; *c*_0_- concentration of investigated compound; α - charge transfer coefficient (accepted being equal 0.5); *E*- electrode potential; parameter θ was introduced to simplify the final formula relating *E* and *R*_ct_.
  1. Copy the first column of values (potential values) in the same sheet in column D.
  2. Enter the constant values of *F*, *c*_0_, *z*, *R*, α, *T*, *E*_0_, *k*_0_ enlisted above into cells C1:C8. Use values *E*_0_ = 0, *k*_0_ = 1∙10^−5^.
  3. Enter formula (2) to calculate θ into cell E1: =EXP($C$1*$C$3/($C$4*$C$6)*(D1-$C$7)).
  4. Copy the formula into cells E2:E21 by selecting E1, clicking **Copy**, selecting range E2:E21 and clicking **Paste**.
  5. Enter formula (1) into cell F1: = $C$8*$C$1^2*$C$3^2/($C$4*$C$6)*$C$2*E1^(1-$C$5)/(1+E1).
  6. Copy the formula into cells F2:F21 by selecting F1, clicking **Copy**, selecting range F2:F21 and clicking **Paste**.
  7. Left click on the graph built at step 5.1, choose **Choose data**, then **Add** and add new data set by specifying entering **D1:D21** as x range and **F1:F21** as y range. NOTE: Two graphs: experimental and simulated automatically marked by different colors will appear on one coordinate plot.
3. Optimize the theoretical function (1) in order to fir experimental data by varying values of equilibrium potential (*E*^0^) and standard rate constant (*k*^0^), being the target parameter. ​NOTE: Change of the values in cells C7 (E0) and C8 (k0) would immediately cause change of the simulated graph.
  1. Change values in cells C7 and C8 manually in order to achieve equality between experimental and simulated graph. NOTE: Change of *E*_0_ moves the bell-like curve along the *x* axis. Change of *k*_0_ controls the height of the bell-like curve. Thus, varying those two only parameters can be used to find a theoretical model corresponding to experimental results (Figure 6). Parameter α (1) controls symmetry of theoretical peak. However, in real systems asymmetry may be caused by the occurrence of side-process rather than by α. Since it influences resulting *k*_0_ value we recommend not to manipulate α value and leave it to equal 0.5.

## Representative Results

The first step is cyclic voltammetry characterization presented in Figure 1. Application of EIS was successful when compounds underwent the fast reversible electrochemical process. Such behavior was often not observed for organic compounds but organic compounds that possess electroconductivity in a solid state was found to be a good specimen for electrochemical kinetic investigation. One such organic compound is shown in the inset of Figure 1.

Registration of impedance spectra was carried out according to the experimental setup (Figure 2), and typical raw resulting data are shown in Figure 3. Analysis of impedance spectra was carried out using special software^24^. The window of the open access program EIS Spectrum analyser^24^ during results processing is shown in Figure 4. An EEC used to fit the spectrum is built manually in the right upper sub-window. The calculated EEC parameters (resistances R1 and R2, capacitance C1 and diffusion impedance parameter W1) are shown in a table in the left upper sub-window. The graph in lower left sub-window illustrates fitting of experimental results (red points) with the theoretically calculated data plot (green line).

Several different EEC may fit experimental spectrum depending on the processes that take place on the electrode surface and their rates (Figure 5). The simplest semi-infinite Warburg element can be used as there is no distortion of solution (*e.g.* rotating of the electrode mixing) and no electrode coating limiting the diffusion. In case of considerably fast electrochemical reactions, resistance R3 (Figure 5**A**) was high enough to be neglected in comparison with other parallel branches of EEC (Figure 5**B**). Moreover, when rate of charge transfer (R2) is significantly higher than diffusion, the charge transfer step becomes limiting and an even simpler EEC (Figure 5**C**) describes the system.

The series resistor R1 is always present in EEC. It corresponds to the external resistance including connectors and solution, except electrode-surface interface. Capacitor C1 characterizes a double layer formed at the electrode interface. The branch including resistor and Warburg element diffusion impedance (Figure 5**A**) corresponds to a fast electrochemical process including two stages: kinetic and diffusion, respectively. The third resistor corresponds to a slower electrochemical process that takes place on the electrode surface and involves solvent or molecules that have undergone fast oxidation or reduction. In some cases, parameters, R3 and W1 were impossible to estimate. Then they might be considered as absent and not taken into account as Figure 5B and **5C** show.

Although EIS provides an estimation of several parameters, the target element which is considered in this work is charge transfer resistor R2 usually assigned as *R*_ct_ in literature^17^,^18^,^19^, which stands in parallel to the capacitor and in series to Warburg element. Its dependence on voltage is shown in Figure 6.

According to the theory of electrochemical kinetics (Protocol, step 5.2), charge transfer resistance is directly related to the standard electrochemical rate constant. Even though matching between experimental and theoretical results was not ideal, it allowed estimation of the value of the standard electrochemical rate constant and defined the value of equilibrium potential by maximum position.

**Figure:**
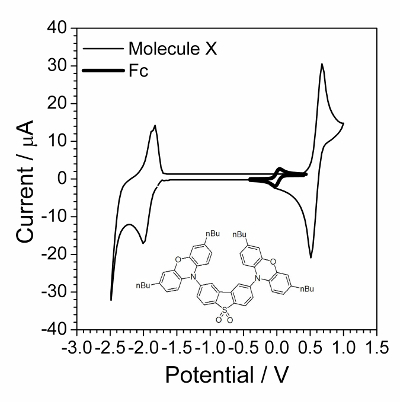

Figure 1**: Cyclic voltammogram of investigated compound overlapped by cyclic voltammogram in presence of small amount of ferrocene.** Solution: 1.0 mol∙L^−1^ Bu_4_NBF_4_ and 0.01 mol∙L^−1^ X in dichloromethane. Structure of compound X (2,8-bis(3,7-dibutyl-10*H*-phenoxazin-10-yl)dibenzo[*b,d*]thiophene-*S,S*-dioxide) is shown in the inset. Please click here to view a larger version of this figure.

**Figure:**
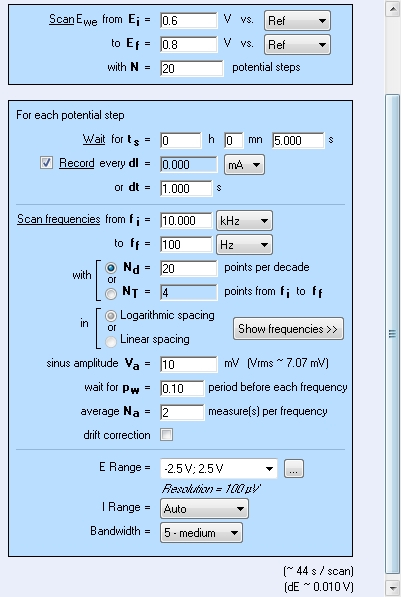

Figure 2**: Experimental setup controlling registration of 20 spectra within the voltage range from 0.6 to 0.8 V in the frequency range from 10 kHz to 100 Hz with 20 points for each decade.** E_i_, E_f_- initial and final potentials respectively, N - number of steps, t_s_- waiting time before each measurement, dt - record time interval, f_i_, f_f_- initial and final frequency, N_D_- number of frequency points in one spectrum, V_a_- ac amplitude, pw - part of time in respect to one point registration used to switch to another frequency, N_a_- number of measurements at one frequency, E range, I range, Bandwidth - technical parameters. Please click here to view a larger version of this figure.

**Figure:**
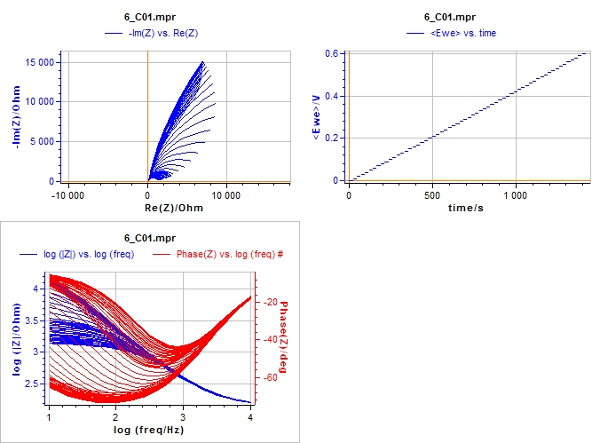

Figure 3**: Scan of screen during impedance spectra registration.** Upper right sub-window: staircase dependence of electrode potential on time. Upper left sub-window: Nyquist plot, imaginary impedance (ordinate), real impedance (abciss). Lower left sub-window: Bode plot, impedance module (left scale), phase shift (right scale), frequency (horizontal scale). Please click here to view a larger version of this figure.

**Figure:**
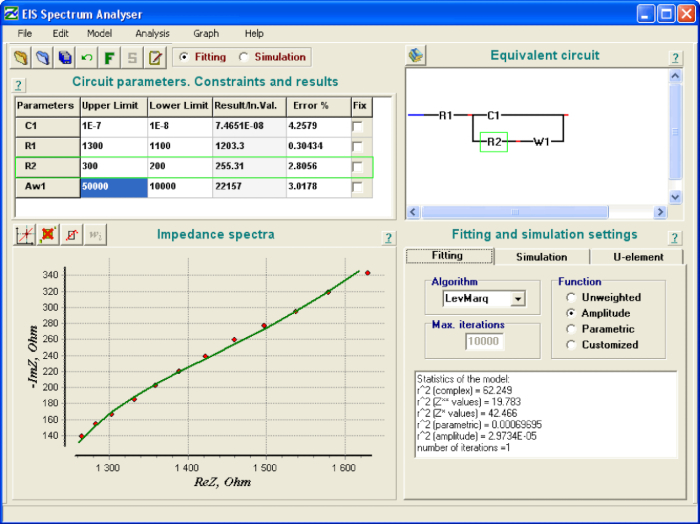

Figure 4**: «EIS Spectrum analyzer» program window during results processing.** Upper left sub-window: parameter values table: C1 - capacitance, R1, R2 - resistances, W1 - Warburg element; lower left sub-window: experimental (green points) and theoretical model (red line) spectra; upper right sub-window: equivalent electrical circuit; lower right sub-window: calculated statistics of fitting. Please click here to view a larger version of this figure.

**Figure:**
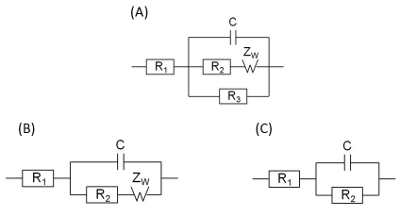

Figure 5**: Equivalent electrical circuits found to fit the impedance spectra of redox processes on the electrode surface.** (A) - reversible electrochemical process accompanied by parallel irreversible process, (B) - reversible electrochemical process, (C) - electrochemical process with kinetic limitation stage. Please click here to view a larger version of this figure.

**Figure:**
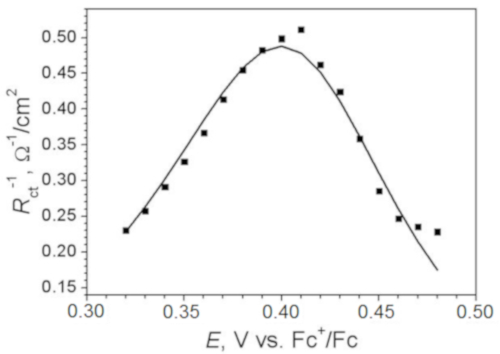

Figure 6**: Inverse values of charge transfer resistance estimated from EIS versus electrode potential.** The line depicts theoretically predicted dependence according to formula (2).

## Discussion

This part of the work will be devoted to an explanation of chosen experimental conditions and discussion of possible applications of the method presented.

Analysis of impedance spectrum may be performed by various software. Here the basic recommendations for EEC analysis method are discussed. One needs to know that there are numerous fitting algorithms and various ways of error estimation. We present an example of using open access software developed by A. Bondarenko and G. Ragoisha^24^ (Figure 4).

Exact estimation of *R*_ct_ value was the main objective of the work. One of the reasons for the choice of the experimental conditions was an intention to conceal the impact of diffusion. Thus, the solution concentration had to be as high as possible. While acquiring the experimental results shown here, the concentration was limited due to economic reasons. The range of frequencies from 10 kHz to 100 Hz was chosen to eliminate the effect of diffusion as well. Diffusion impedance is inversely proportional to the frequency while resistance is not dependent on the frequency. The effect of resistance in the high-frequency part of the spectrum was higher than in the low-frequency part. Spectra were not registered at the frequencies lower than 100 Hz because these data would be useless for resistance calculation. All the electrochemical results obtained in non-aqueous solvent are presented versus ferrocene-oxidized / ferrocene coupled equilibrium potential. For this reason, steps 2.3 - 2.5 are performed.

We considered EIS application to organic molecules characterization. Analysis of other EEC parameters and their potential dependencies in perspective may lead to the revelation of other effects and electrochemical characterization of compounds in solution. Estimation of redox rate constants is useful for describing the kinetics of electroactive compound reduction or oxidation and predicting material behavior in oxidizing or reducing medium.

## Disclosures

The authors have nothing to disclose.
